# Pars plana vitrectomy in patients aged 85 years and older: a single-centre, retrospective cohort study

**DOI:** 10.1007/s10792-023-02891-z

**Published:** 2023-10-18

**Authors:** Julio J. Gonzalez-Lopez, Maria E. Arruza Santos, Jorge Leon Garcia

**Affiliations:** 1 Ophthalmology Department, Hospital Universitario Ramón y Cajal, IRYCIS, Carretera de Colmenar Km 9, 100, 28034 Madrid, Spain; 2 Surgery Department, Universidad de Alcala School of Medicine, Madrid, Spain

**Keywords:** Geriatrics, Ophthalmology, Post-operative complications, Retina, Vitrectomy, Vitreoretinal surgery

## Abstract

**Purpose:**

To describe the epidemiology, indications and surgical results of pars plana vitrectomy (PPV) in patients over 85 years of age.

**Methods:**

A retrospective cohort study was performed including all consecutive patients aged 85 years or older who underwent PPV between September 2018 and March 2022 in a single hospital in Madrid, Spain. Data on diagnosis, comorbidities, surgical indication, surgical details, surgical complications and surgical outcomes were collected from medical records.

**Results:**

A total of 124 eyes of 119 patients (56 males, 47.1%) underwent PPV. Median age was 87 years (range 85–96). The most common surgical indications were complications of cataract surgery in 34 patients (28.6%), macular epiretinal membrane in 32 (26.9%), and rhegmatogenous retinal detachment (RRD) in 12 (10.1%). Mean preoperative best corrected visual acuity (BCVA) was 13.33 ± 42.34 ETDRS letters and improved to 40.05 ± 41.04 letters at 3 months (*p* < 0.001). BCVA had improved in 68.82% of patients at 3 months. Patients with chronic kidney disease (CKD; *p* < 0.001), RRD (*p* = 0.003), ocular trauma (*p* = 0.001) and age-related macular degeneration (AMD; *p* = 0.002) showed worse BCVA at 3 months from surgery. Patients with better preoperative BCVA (*p* < 0.001), and those who underwent 25G PPV (*p* = 0.041) showed better visual outcomes.

**Conclusions:**

PPV is an effective technique for improving visual acuity in patients aged 85 years and older with vitreoretinal diseases. Visual outcomes were better when patients had a better preoperative visual acuity and underwent 25G PPV. Patients with a previous diagnosis of AMD or CKD, and those undergoing surgery for ocular trauma or RRD had worse visual outcomes.

## Introduction

The development of new drugs, surgical techniques and improved preventive medicine after the Second World War and during the *Pax Americana* [1], led to declining mortality rates and an increase in the proportion of people surviving to old age. In 2015, of the estimated 7.3 billion worldwide, 617.1 million (9%) were aged 65 and older. By 2030, the older population will be about 1 billion (12% of the projected total world population), and by 2050, 1.6 billion (17%) of the total population of 9.4 billion will be 65 and older [2]. As life expectancy increases, involutional and chronic eye diseases account for a large proportion of ophthalmic disease, and the geriatric population is becoming a significant part of the patient burden in healthcare practice [3].

Pars plana vitrectomy (PPV) has been widely used for the treatment of various vitreoretinal pathologies since the 1980s. The number of patients over 85 years of age undergoing PPV surgery has increased significantly and is expected to continue to increase in the future, due to the longer life expectancy. Some regions, such as Madrid, have particularly high life expectancy, highlighting the importance of population ageing [4, 5].

The most common indications for PPV are retinal detachment, proliferative diabetic retinopathy, epiretinal membrane (ERM), macular hole and complications of cataract surgery. The incidence of complications associated with PPV has decreased in recent years due to technological advances and increased subspecialisation of vitreoretinal surgeons [6]. However, age-related ocular and systemic comorbidities may increase the rate of complications and compromise visual prognosis in very elderly patients. Few studies have previously focused on the safety and efficacy of PPV in this age group [7–9]. Most importantly, these studies have not comprehensively analysed the influence of systemic, operative and perioperative factors on safety and efficacy outcomes.

The main objective of this study is to describe the surgical indications, operative and perioperative variables and visual outcomes obtained in patients over 85 years of age undergoing PPV. Secondary objectives were to analyze factors associated with visual outcomes and complications in these patients.

## Subjects and methods

A retrospective cohort study was performed, including all patients aged 85 years or older who underwent any vitreoretinal surgery between September 2018 and March 2022 in a single tertiary care, university-affiliated hospital in Madrid, Spain. The protocol, number 104/22, was approved by the Research Ethics Committee of the Ramón y Cajal University Hospital, and all research adhered to the tenets of the Declaration of Helsinki. Informed consent was waived by the Ethics Committee due to the retrospective nature of the data retrieval.

All surgeries were performed with the Constellation Vision System (Alcon, Forth Worth, TX, USA) by experienced ophthalmic surgeons.

### Inclusion and exclusion criteria

All patients aged 85 years and older who underwent PPV at the Ramon y Cajal University Hospital between September 2018 and March 2022 were included in the study. Only one eye per patient was included in the analysis. For patients who underwent surgery on both eyes during the study period, only the first eye was included in the analysis.

Patients who were lost to follow-up during the first month after surgery were excluded from the analysis, unless the loss to follow-up was due to death.

Epidemiological data (including sex, age, and laterality) and clinical data (including best corrected visual acuity (BCVA) at presentation and at 1, 3, and 12 months after surgery, ocular and systemic comorbidities, surgical indication, surgical details, surgical complications, and post-operative systemic events requiring hospitalisation) were collected from the patients' electronic medical records.

### Statistics

All BCVA values were converted from Snellen decimal ratios to Early Treatment Diabetic Retinopathy Study (ETDRS) LogMAR letters at all time points. According to Lange and collaborators[10, 11], visual acuities below measurable ETDRS letters were converted to − 15 letters, − 30 letters and − 45 letters for counting fingers, hand movements and light perception, respectively. Visual improvement/worsening was defined as a change of at least ± 5 ETDRS letters.

Data were entered into a computerised database. Qualitative variables were expressed as percentages. Quantitative variables were expressed as mean values (± standard deviation [SD]) if they followed a normal distribution or as median values (interquartile range) if they did not. Differences between paired variables were tested using paired *T* test when normality conditions allowed. Otherwise, the Wilcoxon test was used. Multivariate analysis was carried out using multiple linear regression models. The selection of variables in the final model was performed using a forward-conditional method, with significance levels of ≤ 0.05 for inclusion and ≥ 0.1 for exclusion. *R*^2^ was used to assess the overall model fit. The validity of the model was assessed using the bootstrap technique[12], for which 5000 computer-generated samples, each including 93 patients, were derived from the study population by random selection with replacement. For each bootstrap sample, the primary model was re-fitted and the average beta was obtained. Data were analysed using IBM SPSS Statistics, version 23.0 for Unix (International Business Machines Corporation, Armonk, NY, USA).

## Results

**Fig. 1:**
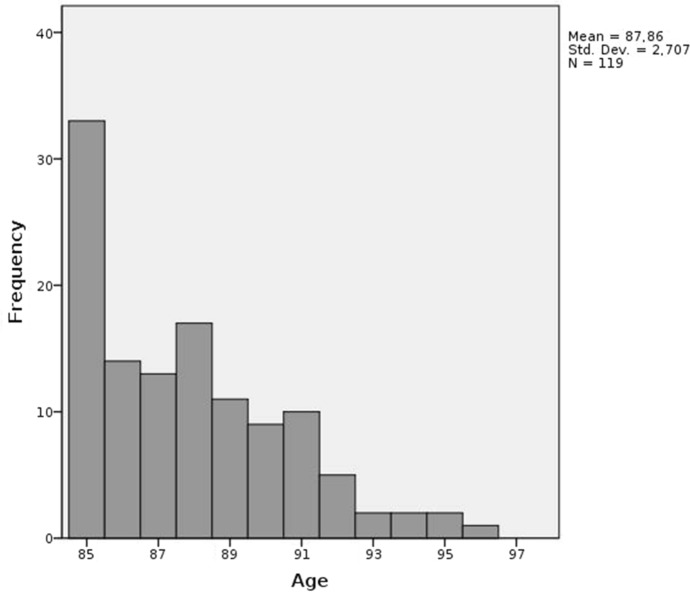
Age distribution of the 119 patients aged 85 years and older who underwent pars plana vitrectomy during the study period

During the study period, 124 eyes of 119 patients (56 males, 47.1%) underwent PPV. Figure 1 shows the age distribution of the patients included in the study. The median age was 87 years (range 85–96 years). Thirty-one patients were aged 90 years or older.

### Underlying vitreoretinal disease

The right eye was operated on in 58 cases (47.1%). Table 1 shows the surgical indications for the included patients. The most common surgical indications were complications of cataract surgery (34 patients, 28.6%), macular epiretinal membrane (32 patients, 26.9%) and rhegmatogenous retinal detachment (RRD; 12 patients, 10.1%). Other less frequent indications were massive submacular haemorrhages secondary to age-related macular degeneration (AMD), full thickness macular hole, endophthalmitis and ocular trauma (9, 5, 4 and 4 patients respectively). Twenty-one eyes were phakic (17.6%), 34 eyes were aphakic (28.6%), and the rest (64 eyes; 53,8%) were pseudophakic. Of the 21 phakic eyes, all of them presented significant cataracts.

**Table 1:** Distribution of indications for pars plana vitrectomy and change in best corrected visual acuity (BCVA) in ETDRS letters for each indication

Indication	*N* = 119	Percentage	Median preoperative BCVA (range)	Median BCVA at 3 months (range)	Median change in BCVA	*p*
Dropped IOL/retained lens fragment	34	28.6	− 15 (− 45–80)	70 (− 45–80)	40 (− 110–106)	0.002
Epiretinal membrane	32	26.9	60 (− 15–75)	70 (− 15–82)	7.5 (− 15–60)	0.002
Submacular haemorrhage/AMD complications	15	12.6	− 15 (− 30–35)	32.5 (− 30–65)	37.5 (0–80)	0.005
Rhegmatogenous retinal detachment	14	11.8	− 30 (− 45–80)	− 15 (− 45–75)	0 (− 5–53)	0.104
Full-thickness macular hole	5	4.2	50 (− 15–60)	50 (50–70)	5 (0–75)	0.180
Proliferative diabetic retinopathy	4	3.4	22.5 (− 30–65)	27.5 (− 15–70)	10 (− 80–100)	0.655
Bacterian endophthalmitis	4	3.4	− 5 (− 30–30)	57.5 (20–65)	57.5 (0–95)	0.102
Ocular trauma	4	3.4	− 45 (− 45–− 45)	− 30 (− 45–− 15)	15 (0–30)	0.317
Non-diabetic vitreous haemorrhage	7	5.9	− 15 (− 30–50)	55 (− 30–76)	47.5 (0–100)	0.043

### Ocular and systemic comorbidities

Table 2 shows the frequency of observed ocular and systemic comorbidities in the included patients. The most common ocular condition observed in the operated eyes was glaucoma (23 patients, 19.3%), followed by cataract in 21 (17.6%) and AMD in 20 (16.8%). Only 54 patients (45.4%) had no ocular comorbidity.

**Table 2:** Ophthalmic and systemic comorbidities among patients aged 85 years and older undergoing pars plana vitrectomy

Comorbidities	*N*	Percentage
**Ocular (any)**	**65**	**54.6**
Glaucoma	23	19.3
Cataract	21	17.6
Age-related macular degeneration	20	16.8
Diabetic macular oedema	7	5.9
Retinal vein occlusion	4	3.4
**Systemic (any)**	**112**	**94.1**
High blood pressure	105	88.2
Dyslipidemia	84	70.6
Diabetes mellitus	39	32.8
Heart failure/ischaemic heart disease	24	20.2
Chronic kidney disease	18	15.1
Cerebrovascular disease	16	13.4
Chronic obstructive pulmonary disease	15	12.6
Dementia	7	5.9

The most common systemic disease was hypertension in 105 patients (88.2%), followed by dyslipidaemia in 84 (70.6%) and diabetes mellitus in 39 (32.8%). Only 7 patients (5.9%) had no relevant medical condition.

### Operative and perioperative parameters

Four patients (3.4%) required perioperative hospitalisation. Anaesthesia was local (retrobulbar or subtenon) in 115 patients (96.6%) and general in 4. 23G PPV was performed in 22 patients (18.5%), 25G in 87 (73.1%) and 27G in 10 (8.4%). Combined surgery with phacoemulsification, intraocular lens implantation and vitrectomy was performed in 20 (16.8%) out of 21 phakic patients undergoing vitrectomy.

### Visual results

**Fig. 2:**
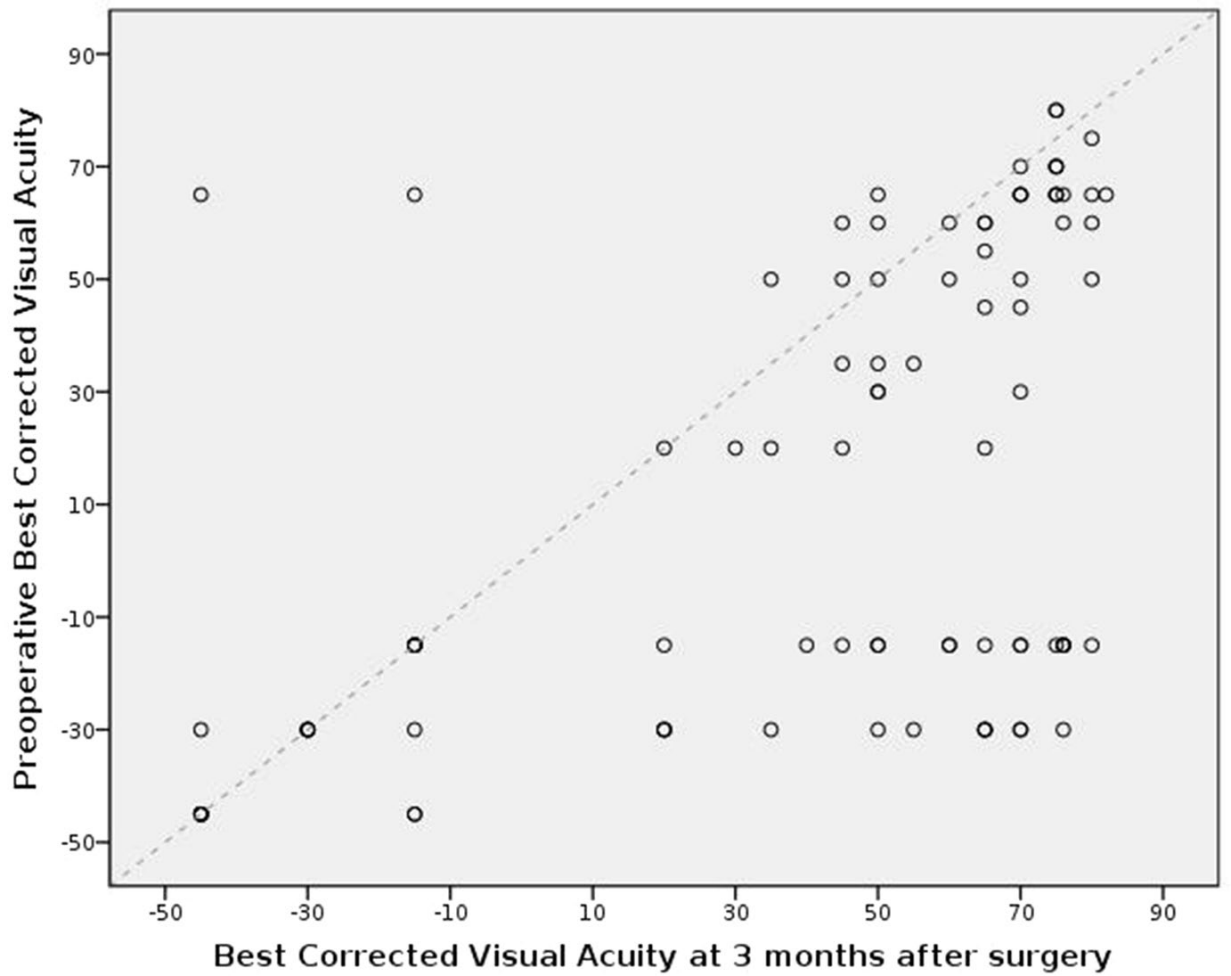
Dot plot representing the best corrected visual acuity in ETDRS letters change from preoperative to 3 months post-operative in the 94 patients who completed at least 3 months of follow-up

Due to the effect of tamponing gases on vision, visual results were analysed only in the 93 patients who completed at least 3 months of follow-up (after their last surgery if they required more than one surgery on the study eye within the first 3 months). Figure 2 shows the visual acuity at 3 months in the 93 patients. The mean preoperative best corrected visual acuity was 13.33 ± 42.34 ETDRS letters and improved to 40.05 ± 41.04 letters at 3 months (*p* < 0.001). After 3 months, best corrected visual acuity decreased in 10 patients (10.75%), remained stable in 19 patients (20.43%) and improved in 64 patients (68.82%). Table 1 summarises the visual outcomes by indication for surgery.

Table 3 shows the results of the multiple linear regression model predicting final best corrected visual acuity at 3 months. Patients with chronic kidney disease (CKD; *p* < 0.001), rhegmatogenous retinal detachment (*p* = 0.003), ocular trauma (*p* = 0.001) and AMD (*p* = 0.002) had worse BCVA 3 months after surgery. Patients with better preoperative BCVA (*p* < 0.001) and those who underwent 25G PPV (*p* = 0.041) showed better visual outcomes. Bootstrap validity assessment confirmed that all these factors were independent predictors of final BCVA in patients aged 85 years and older undergoing PPV, with the exception of 25G vitrectomy (*p* = 0.066).

**Table 3:** Multiple linear regression model for factors predicting final ETDRS best corrected visual acuity (BCVA) in patients aged 85 years and older undergoing pars plana vitrectomy (*N* = 93; *R*^2^ = 0.525), and results of bootstrap multiple linear regression analysis for factors predicting final ETDRS best corrected visual acuity (BCVA) in patients aged 85 years and older undergoing pars plana vitrectomy (*N* = 93; 5000 random generated samples)

Variable	*β*	Standard error of *β*	Standardised *β*	*p*	*β*_bootstrap_	Standard error of *β*_bootstrap_	*p*_bootstrap_
Comorbidity: Kidney failure	− 37.722	8.924	− 0.330	< 0.001	− 37.722	10.021	< 0.001
Indication: Rhegmatogenous retinal detachment	− 29.658	9.845	− 0.235	0.003	− 29.658	10.435	0.008
Indication: Ocular trauma	− 72.084	21.914	− 0.256	0.001	− 72.084	13.316	< 0.001
Comorbidity: age-related macular degeneration	− 26.275	8.166	− 0.249	0.002	− 26.275	7.461	0.002
ETDRS BCVA on presentation	0.303	0.080	0.313	< 0.001	0.303	0.091	0.002
25G pars plana vitrectomy	14.337	6.896	0.159	0.041	14.337	7.633	0.066
Constant	41.379	6.489		< 0.001	41.379	7.640	< 0.001

### Safety analysis

Twenty-three patients (19.3%) had post-surgical complications: 9 retinal detachments (3 of them redetachments), 5 choroidal detachments, 7 macular oedema and 2 endophthalmitis. Ten patients (8.4%) required more than one vitreoretinal procedure. One patient (0.8%) required evisceration after retinal redetachment with choroidal bleeding and intractable pain despite analgesia.

The only patient who remained phakic after vitrectomy developed cataract progression requiring phacoemulsification and primary IOL implantation 6 months after vitrectomy.

Eight patients (6.7%) required hospitalisation in the month following surgery (3 ischaemic stroke, 2 pneumonia, 1 acute cholangitis, 1 heart failure and 1 hyperglycaemic decompensation). In two of the three post-operative ischaemic cerebrovascular accidents, the patient was previously on clopidogrel and was switched to acetylsalicylic acid one week before surgery. One patient developed coronavirus disease 2019 (COVID-19) pneumonia and died in the month following surgery. No statistically significant associations were observed between post-operative admissions and any operative or perioperative parameters.

## Discussion

The population of very elderly patients, aged 85 years and older, has been steadily increasing worldwide and particularly in western societies [13]. This has raised concerns about the efficacy and safety of surgical interventions, given the comorbidities and frailty of these patients [7].

Compared to younger patients, the indications for PPV in elderly patients are generally similar, but show some small differences. On the one hand, some indications such as submacular haemorrhage, dislocated IOL or dislocated nucleus are more common, which is not surprising as the prevalence of AMD and cataract increases with age. This observation is in agreement with previous series [7–9]. On the other hand, severe vitreoretinal diseases such as ocular trauma and endophthalmitis are also overrepresented. This has also been observed previously [7, 8] and may be explained by patient frailty. Not only are older patients more likely to fall, but they are also more likely to suffer from more severe injuries when they do fall [14]. In addition, older patients are known to be at a higher risk of endophthalmitis following procedures such as cataract surgery compared to younger patients [15].

Proliferative diabetic retinopathy represented a lower percentage of the surgical cases compared to younger cohorts [16]. This is consistent with findings in previous series [7, 8]. A possible explanation for this is that good general health is required to survive to 85 years and older (survival bias). Patients who live to 85 years or older are less likely to have poorly controlled diabetes mellitus. This possible survival bias has also been observed for other complications of diabetes mellitus [17]. It is also important to note that diabetic patients in Madrid have been shown to have better glycaemic control and a lower incidence of diabetic retinopathy [18], which could also lead to a less frequent need for vitrectomy in our series.

A statistically significant improvement in post-operative BCVA was observed for the overall cohort, and for individual indications such us dislocated IOL/retained lens fragments, epiretinal membrane, submacular haemorrhage/AMD complications and non-diabetic vitreous haemorrhage. Although an improvement in median post-operative BCVA was observed for the remaining indications, the results did not reach statistical significance, probably due to the small sample size. Interestingly, the improvement in BCVA after vitrectomy for dreaded complications of AMD such as submacular haemorrhages is significant and similar to that observed in younger cohorts [19].

Multivariable statistical analysis in our cohort revealed some interesting findings. Firstly, patients with a previous diagnosis of AMD had lower final visual acuity. This was also found in the series by Anteby and colleagues [7], and confirms that a prior diagnosis of AMD is a negative prognostic factor for improvement in BCVA when considering vitrectomy for all indications in patients over 85 years of age.

Second, patients with CKD also showed worse visual outcomes after PPV. CKD and ocular disease share common risk factors and pathogenic mechanisms, with the renin–angiotensin–aldosterone system as the main player [20]. Patients with CKD are more prone to retinal comorbidities such as diabetic retinopathy [21] and AMD [22]. However, these potential confounders were adjusted for in the multivariate analysis. Thus, our data suggest that CKD is another independent negative prognostic factor for visual improvement after pars plana vitrectomy in patients aged 85 years and older.

Thirdly, patients aged 85 years and older who underwent vitrectomy for retinal detachment repair and ocular trauma had worse visual outcomes at 3 months than patients of the same age who underwent vitrectomy for other indications. Eyes with both retinal detachment [23] and ocular trauma [24] are ophthalmic emergencies with a significant risk of visual sequelae if not treated promptly. We hypothesise that patients aged 85 years and older may delay their presentation to the ophthalmic emergency service, thereby compromising their visual outcomes. This delay may be due to various comorbidities such as mobility problems, sensory deficits or cognitive impairment, which are more common in older age [25]. Due to the retrospective nature of the study, we could not analyse the time between symptom onset and vitrectomy, as this may be a significant predictor of visual outcome in these indications. Therefore, it is important to educate general practitioners and the general population about prompt referral to an ophthalmic emergency service in cases of sudden visual loss, regardless of the patient’s age.

Finally, preoperative BCVA and 25-gauge vitrectomy were positive, independent prognostic factors for post-operative BCVA. Preoperative BCVA is known to be a good predictor of final BCVA in several ocular diseases, including AMD-related submacular haemorrhages [19], retinal detachment [26, 27], ocular trauma [28, 29] and uveítis [5]. The positive prognostic factor of 25-gauge vitrectomy in patients aged 85 years and older may be due to a lower risk of post-operative hypotony [30, 31] or iatrogenic retinal breaks [32]. However, this finding should be treated with caution, as the protective factor of 25-gauge vitrectomy did not pass the validity assessment of the multivariate model (*p* = 0.066).

The ophthalmological complications observed after PPV are similar to those in younger patients, although the complication rates are slightly higher than in their younger counterparts. Redetachment rates after PPV for retinal detachment repair have been shown to be lower in patients aged between 20 and 79 years [33]. Our observation is consistent with these findings. This may be due to increased difficulty in maintaining post-operative indications and posture in very elderly patients, or to delayed presentation due to comorbidity. Of the 3 retinal redetachments in our cohort, two were chronic retinal detachments with a long history of visual loss in the study eye. One of the redetachments eventually required evisceration due to massive choroidal bleeding and uncontrollable pain. These findings highlight once more the importance of prompt referral of cases of sudden visual loss in the elderly, as delayed presentation can lead to not only to blindness, but also to pain and the need for more aggressive surgery. In addition, vitreoretinal surgeons should be aware of this risk when performing “heroic surgery” on eyes with a very poor visual prognosis in the elderly, as “skilful neglect” [34] may be a preferable option in some cases, especially when the fellow eye remains healthy and useful.

Older age has also been described as a risk factor for endophthalmitis after cataract surgery in the past [15]. Our results suggest that this may also be the case after PPV. We hypothesise that very elderly patients may have more difficulty in complying with post-operative care instructions, thus reducing the efficacy of prophylactic measures such as post-operative topical antibiotherapy.

Systemic events were rare in our cohort. The only case of perioperative death observed was a case of COVID-19 pneumonia during the second wave of COVID-19 in Madrid, which took place between July and November 2020 [35], before vaccination became available on 27th December 2020 [36]. In addition, we observed 3 cases of ischaemic cerebrovascular disease within 1 month after PPV. Anteby and colleagues also describe 2 cases of cerebrovascular disease between 5 days and 5 months post-operatively in their series [7]. Interestingly, 2 out of 3 patients in our series had been switched from clopidogrel to acetylsalicylic acid one week before surgery. Mason and colleagues evaluated the risk of surgical complications in a series of 118 patients on clopidogrel undergoing 25-gauge PPV, and found no anaesthesia-related haemorrhagic complications, and a similar rate of transient vitreous haemorrhage in the clopidogrel and control groups [37]. In addition, Huang and colleagues described a lower risk of recurrent stroke in patients aged 80 years and older treated with clopidogrel compared with those treated with acetylsalicylic acid [38]. Therefore, we recommend that elderly patients on clopidogrel undergoing PPV should continue their current therapy without stopping or switching.

The main limitation of this study is its retrospective nature. However, as the preoperative assessment and surgical protocols are comprehensively recorded in the medical records, most of the collected data were complete for all included patients. A second limitation is the lack of a control group including younger patients, which could help to better highlight the differences in surgical outcomes of vitreoretinal surgery in patients aged 85 years and older. Future studies comparing surgical outcomes of PPV in different age groups are needed to confirm our results. Finally, there is a possibility of a selection bias, as patients considered too frail to undergo surgery, with very poor visual prognosis or those who refused surgery themselves were excluded from the cohort.

Despite these limitations, the study is relevant as it is, to the best of our knowledge, the largest cohort of patients over 85 years of age undergoing PPV. In addition, it is the first to analyze the impact of surgical factors such as vitrectomy gauge, and systemic comorbidities such as CKD on the visual prognosis.

In conclusion, our study suggests that PPV is an effective and safe technique for improving visual acuity in patients aged 85 years and older with vitreoretinal disease, and can be performed on an outpatient basis and under local anaesthesia in the vast majority of patients. Visual outcomes were better if the patient had a better preoperative visual acuity and underwent 25G PPV. Patients with a previous diagnosis of AMD or CKD, and those undergoing surgery for ocular trauma or rhegmatogenous retinal detachment showed poorer visual outcomes.

## Data Availability

The data that support the findings of this study are not publicly available due to patient privacy and ethical restrictions. However, they can be shared upon reasonable request from the corresponding author, and after the approval from the Research Ethics Committee of the Ramón y Cajal University Hospital.
